# The effects of psychosocial aftercare following pediatric chronic pain treatment withstand the coronavirus disease 2019 pandemic: long-term outcomes of a randomized controlled trial

**DOI:** 10.1097/PR9.0000000000001226

**Published:** 2024-12-24

**Authors:** Lisa-Marie Rau, Meltem Dogan, Gerrit Hirschfeld, Markus Blankenburg, Michael C. Frühwald, Rosemarie Ahnert, Sarah Braun, Ursula Marschall, Boris Zernikow, Julia Wager

**Affiliations:** a German Paediatric Pain Centre, Children's and Adolescents' Hospital Datteln, Datteln, Germany; b Department of Children's Pain Therapy and Paediatric Palliative Care, Witten/Herdecke University, Faculty of Health, School of Medicine, Witten, Germany; c Faculty of Business, University of Applied Sciences and Art Bielefeld, Bielefeld, Germany; d Paediatric Pain Centre Baden-Württemberg, Department of Pediatric Neurology, Olgahospital Stuttgart, Stuttgart, Germany; e Pediatrics and Adolescent Medicine, Bavarian Children's Pain Center, University Hospital Augsburg, Germany; f Department of Medicine and Health Services Research, BARMER Health Insurance, Wuppertal, Germany; g PedScience Research Institute, Datteln, Germany

**Keywords:** COVID-19, Chronic pain, Pediatrics, Psychosocial aftercare, Longitudinal study, Randomized controlled trial

## Abstract

**Introduction:**

Intensive interdisciplinary pain treatment (IIPT) is the best therapy available for children and adolescents affected by severe chronic pain. Psychosocial aftercare (PAC) offered for 6 months after IIPT can improve treatment outcomes for up to 12 months.

**Objectives:**

The current study is the first to explore whether PAC is superior to treatment as usual at a long-term follow-up of 18 to 33 months after discharge—including when facing the coronavirus disease 2019 (COVID-19) pandemic.

**Methods:**

A multicenter randomized controlled trial investigated the impact of PAC with follow-up assessments in 2021 during the COVID-19 pandemic (N = 209). Multilevel models and regression analyses explored long-term treatment effects on pain characteristics and psychological outcomes, associations of pandemic-related burden with these outcomes, and whether pandemic-related burden moderates those effects.

**Results:**

Patients who received PAC significantly improved treatment outcomes concerning pain characteristics and psychological well-being 18 to 33 months after IIPT discharge. A COVID-19 infection among family members or peers was associated with a lower probability of having chronic pain at long-term follow-up, whereas having missed or rescheduled health care appointments was associated with a higher probability. Positive evaluations of family time during the pandemic were associated with better psychological well-being. Regardless of pandemic-related burdens, PAC had similarly better outcomes than treatment as usual. Only for anxiety, greater burdens mitigated the advantages of PAC.

**Conclusion:**

Despite adverse circumstances, PAC retained its superiority long-term. Integrating PAC into routine health care to support families after IIPT should be prioritized to yield enduring treatment effects, acting as a buffer against the impacts of unpredictable adverse events.

## Abstract 2

Supplemental Digital Content is Available in the Text.

Using psychosocial aftercare to support pediatric patients after chronic pain treatment has long-lasting benefits and mitigates detrimental effects of adverse events, such as a pandemic.

## 1. Introduction

Intensive interdisciplinary pain treatment (IIPT) is an effective therapy for children and adolescents affected by severe chronic pain conditions.^11^ Previous analyses have demonstrated that a newly developed psychosocial aftercare (PAC) program after IIPT improves IIPT effectiveness regarding pain characteristics and psychological well-being up to 12 months after IIPT discharge.^14,15^ Psychosocial aftercare aims to support patients and families in maintaining their individualized therapy goals during the transition from the highly structured, clinician-led IIPT to attaining self-sufficiency in everyday life. Additional support may be particularly relevant to pediatric patients carrying high psychosocial burdens because these patients have a higher risk of IIPT treatment failure.^15^ However, PAC's supplementary effectiveness remains consistent regardless of patient and family characteristics—with the exception of single-parent families, who experience greater benefits from PAC.^15^

During the coronavirus disease 2019 (COVID-19) pandemic, many families dealt with substantially higher psychosocial burden.^3^ Governmental mitigation measures such as lockdowns, school closures, and social distancing imposed significant restrictions on young people and their everyday lives, resulting in notable reductions in social contacts.^3^ Many families experienced financial losses because of unemployment or short-time work.^10,16^ As a consequence, the incidence of mental disorders surged across all age groups.^16,34^ Psychosocial stressors experienced during the pandemic were related to the development of (chronic) pain.^32,34^ During the initial lockdowns, pediatric chronic pain prevalence and severity declined.^2,5,25,26,29,32^ As the pandemic progressed, however, the positive aspects of lockdowns might have faded in light of prolonged uncertainties, disruptions in routine, and social isolation. This could have contributed to the subsequent increases in depression,^37^ suicidality,^35^ and mental health problems^42^ later in the pandemic. Perceiving the pandemic as progressively more stressful might, in turn, negatively affect the success of IIPT treatment.

To date, no pediatric study has explored the efficacy of IIPT long-term, into the second year of the COVID-19 pandemic. By chance, we conducted a randomized controlled multicenter study on the effectiveness of PAC compared with standard IIPT aftercare during the COVID-19 pandemic. In the current study, IIPT took place before the pandemic; the first mandated lockdown came into effect approximately 5 months after the final IIPT enrollment. This study presents long-term follow-up outcome data spanning 18 to 33 months after IIPT discharge. This assessment took place in 2021, during the COVID-19 pandemic.

The current study was designed with 3 primary goals. First, it aimed to explore the treatment outcomes of PAC vs treatment as usual (TAU) during the long-term follow-up period assessed during the COVID-19 pandemic. It was expected that even during the pandemic, PAC would be more effective at reducing pain symptoms and psychological impairment than TAU. The second goal was to identify the association of pandemic-related burdens with the long-term treatment outcomes of IIPT. It was hypothesized that a greater burden would be associated with worse pain and psychological parameters. The third analysis examined whether the link between pandemic-related burden and negative treatment outcome is weaker in the PAC group, indicating a moderation effect.

## 2. Methods

### 2.1. Study design

The current study is part of a multicenter randomized controlled trial evaluating the effect of PAC on children and adolescents who received IIPT at one of 3 specialized pediatric pain centers in Germany. Patients were randomly allocated to the PAC or TAU group (1:1 ratio; stratified by pain center; for more details, see Ref. 14). Patient outcomes were collected at 6 assessments: admission (PRE-IIPT); discharge (POST-IIPT); 3, 6, and 12 months after discharge from IIPT; and 18 to 33 months after IIPT discharge (LONG-TERM). The randomized controlled trial was preregistered in the German Clinical Trials Register (registration-ID: DRKS00015230). For this study, only the first 2 assessments and the LONG-TERM follow-up were analyzed. Results of the other 3 follow-ups have been reported in previous publications.^14,15^

### 2.2. Sample

**Figure 1.**
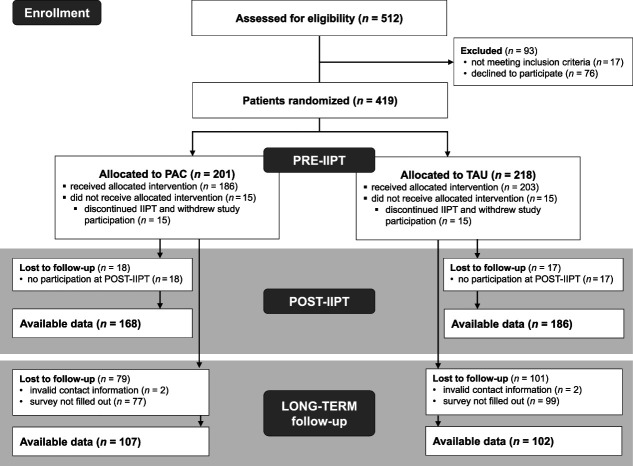
Flowchart of participation. LONG-TERM follow-up took place 18 to 33 months after discharge from IIPT. IIPT, intensive interdisciplinary pain treatment; PAC, psychosocial aftercare; TAU, treatment as usual.

Eligibility criteria included (1) admission for IIPT between September 2018 and October 2019, (2) aged 8 to 17 years, (3) German language proficiency, and (4) informed consent provided by both patients and parents. All patients fulfilled the 11th revision of the International Classification of Diseases (*ICD-11*) chronic primary pain criteria.^48^ Patients were excluded if they withdrew their consent. The sample used in the current analyses includes only patients who completed the LONG-TERM assessment (May–October 2021; interval after discharge: M = 25.9 months, SD = 3.7 months; for characteristics of the total sample, see Ref. 14). The response rate at LONG-TERM was 50% (N = 209; M_age_ = 14.29 years, SD_age_ = 1.99 years; n_girls_ = 154; see Fig. 1 for participant flowchart). Patients participating in LONG-TERM did not differ from those who dropped out in terms of demographic and pain-related characteristics (all *P* > 0.05 after Benjamini–Hochberg correction; Table 1). The PAC (n = 107) and TAU (n = 102) groups did not differ significantly PRE-IIPT (Table 1 includes sample descriptives) nor POST-IIPT. Follow-up duration was similar between both groups (M_TAU_ = 786.79 days, SD_TAU_ = 113.86 days; M_PAC_ = 790.24 days, SD_PAC_ = 112.70 days; *P* = 0.826).

**Table 1:** Descriptive statistics PRE-IIPT for the total sample, sample dropped out at LONG-TERM, and sample with complete LONG-TERM assessment.

	Total sample (N = 419)	Dropout (n = 210)	LONG-TERM (N = 209)*	*P* (dropout)	*P* adjusted (dropout)	PAC (n = 107)*	TAU (n = 102)*	*P* (treatment)
Age	14.29 (2.10)	14.30 (2.21)	14.29 (1.99)	0.987	0.987	14.31 (2.09)	14.27 (1.90)	0.902
Gender (girl)	303 (72%)	149 (71%)	154 (74%)	0.606	0.909	77 (72%)	77 (75%)	0.673
Pain onset				0.093	0.419			0.558
3–6 mo	37 (9%)	13 (6%)	24 (11%)			14 (13%)	10 (10%)	
6–12 mo	85 (20%)	39 (19%)	46 (22%)			21 (20%)	25 (25%)	
1–2 y	69 (16%)	42 (20%)	27 (13%)			15 (14%)	12 (12%)	
2–3 y	79 (19%)	37 (18%)	42 (20%)			18 (17%)	24 (24%)	
>3 y	149 (36%)	79 (38%)	70 (33%)			39 (36%)	31 (30%)	
Maximum pain intensity	8.15 (1.74)	8.01 (1.97)	8.28 (1.46)	0.115	0.345	8.24 (1.54)	8.32 (1.39)	0.691
Average pain intensity	6.07 (1.89)	6.02 (1.96)	6.11 (1.83)	0.642	0.825	6.08 (1.90)	6.14 (1.77)	0.834
Pain self-efficacy	18.04 (8.85)	17.18 (8.90)	18.90 (8.74)	0.047	0.423	18.64 (9.03)	19.18 (8.46)	0.655
Depression symptoms	9.74 (5.54)	9.96 (5.73)	9.53 (5.35)	0.422	0.760	9.89 (5.45)	9.15 (5.24)	0.318
Anxiety symptoms	27.44 (18.19)	27.12 (19.34)	27.76 (16.99)	0.722	0.812	28.24 (16.87)	27.25 (17.19)	0.673
Health-related quality of life	97.38 (15.56)	96.36 (16.21)	98.41 (14.85)	0.179	0.403	98.09 (15.10)	98.74 (14.65)	0.755

Cells contain mean values (SDs) for numeric variables or absolute (relative) frequencies for dichotomous variables. χ^2^ and *t* tests were applied as appropriate (*P* were adjusted for multiple testing using Benjamini–Hochberg correction; R package *compareGroups*).

* Sample included in further analyses.

PAC, psychosocial aftercare.

### 2.3. Procedure

Participating patients were randomized to either the TAU or PAC group; blinding this assignment was not feasible. Participants completed PRE-IIPT and POST-IIPT assessments on tablet computers while at their pain center and LONG-TERM online. During their inpatient stay, all participants received manualized IIPT.^13^ PAC began at the IIPT discharge meeting where the families were introduced to their designated PAC social worker.^14^

### 2.4. Intervention

Patients assigned to receive TAU were offered the standard aftercare consisting of two 1- to 1.5-hour appointments with their treating psychotherapist and pediatrician at their pain center, 3 and 6 months after discharge. Families could arrange more appointments on request.

In addition to the standard aftercare, patients in the PAC group received a manualized intervention based on case management. For up to 6 months after discharge, a trained social worker—in collaboration with a team of psychologists and physicians—supported families with implementing and retaining the recommendations received during IIPT. This included, eg, needs assessment, putting family in contact with other health care providers, and relapse prevention. Tailored to the individual family's needs, patients and parents could decide the mode and frequency of their interactions with the social worker. On average, families were contacted 10 times (range: 2–32). Families mostly chose telephone contacts, and 27% had at least one home visit (for more details see Ref. 14).

### 2.5. Measures

All measures were collected at all 3 assessments, unless indicated otherwise.

#### 2.5.1. Pain characteristics

Patients reported the frequency of pain episodes (0 = once a month or less; 1 = multiple times per month; 2 = once a week; 3 = multiple times per week; 4 = once a day; 5 = multiple times per day; and 6 = always^36^).

Chronic pain was defined as having pain occurring at least once a week during the last 4 weeks to ensure recency of pain. Pain occurring less frequently was classified as no/infrequent pain.^47^

Maximum and average pain intensity within the past 4 weeks was measured on a numerical rating scale (NRS; 0 = no pain and 10 = strongest pain^44^). Patients who did not report any pain within the past 3 months were coded as having “no pain.”

#### 2.5.2. Psychological measures

Pain self-efficacy was assessed using the Scale for Pain Self-Efficacy.^41^ Patients rated the 11 items on a 5-point scale (0 = not true, 4 = true), which are summed to a total score. Higher values indicate stronger perceptions of pain self-efficacy. Only patients experiencing pain completed the scale (PRE-IIPT: all patients; POST-IIPT: n = 189; and LONG-TERM: n = 136). Internal consistency was sufficient across all assessments (Cronbach's α = 0.87–0.96).

To measure emotional distress, symptoms of depression and anxiety were assessed using the Revised Child Anxiety and Depression Scale.^9,40^ The 10 items of the depression and 37 items of the anxiety subscale are summed to total scores (across assessments Cronbach's α_depression_ = 0.85–0.90; α_anxiety_ = 0.94–0.96). Patients responded on a 4-point scale (0 = never and 3 = always), where higher values indicate more symptoms of depression and anxiety.

Health-related quality of life was assessed at PRE-IIPT and LONG-TERM using the Kidscreen-27.^33^ Patients rated the 27 items on a 5-point scale (1 = never, 5 = always), where higher values indicate better quality of life. Ratings were summed to a total score (across assessments, Cronbach's α = 0.91–0.95).

#### 2.5.3. Pandemic-related burden

The individual burden of COVID-19 was assessed at LONG-TERM. The following pandemic-related factors were considered (dichotomous unless otherwise indicated): personal COVID-19 infection (1 item), the presence of long-term medical consequences of COVID-19 (7 deficits: difficulties breathing, tasting, or smelling, fatigue, cough, increased pain, and other symptoms), the occurrence and severity of COVID-19 infections (eg, hospitalization, intensive care admission, and death) in the immediate family and peer network (4 items), and the social ramifications of the pandemic (4 items, including school absence because of personal quarantine or COVID-19 symptoms, pandemic-related barriers to patient's health care utilization, financial burden on the family [11-point NRS], and difficulties organizing child care, such as during school closures [11-point NRS]). In addition, a cumulative score of pandemic-related burden was calculated as the sum of individual items (no burden = 0 and burden present = 1), with the exception of items assessed using an NRS, where ratings ≥3 were scored as 1, with possible total scores ranging from 0 to 16.

Patients also reported how often during the pandemic they experienced time spent with their family as tense, hectic, harmonious, or relaxed on a 5-point scale (0 = never and 4 = always^32^).

### 2.6. Data analyses

Comparative analyses of pain-related and psychological treatment outcomes were performed between the PAC and TAU groups. χ^2^ tests were used for chronic pain analyses. For continuous variables, mixed model analyses were deployed (analyses of variance using multilevel model comparisons with implemented autocorrelative covariance structure; R package *nlme*^30^). Both time and treatment group were included as categorical predictors in all models. Regarding time, LONG-TERM was compared with the reference categories of PRE-IIPT (for assessing overall treatment effect) and POST-IIPT (to evaluate the intervention effect). For treatment group, TAU was designated as the reference category. The time × group interaction indicates that the 2 groups diverge over time.

The sample's pandemic-related burden is presented using descriptive statistics and compared between PAC and TAU (using χ^2^ and *t* tests). To control for multiple testing and identify the most relevant COVID-19–related predictors associated with LONG-TERM chronic pain and psychological parameters, least absolute shrinkage and selection operator (LASSO) regressions were conducted (R package *glmnet*^20^). The regularization-parameter lambda was chosen based on cross-validation, following the “one-standard-error rule.”^23^ To minimize bias, only COVID-19 variables with at least 10 patients scoring above 0 were investigated as predictors in the models. Age, gender, condition, and duration of follow-up interval were consistently integrated into each model as control variables. To facilitate comparison with binary predictors, each continuous predictor was centered and divided by twice the SD.^21^ The associations of LONG-TERM chronic pain and psychological parameters with the total pandemic-related burden were explored through regression analyses. Besides the main effect (ME) of treatment group, the burden × group interactions are considered in each regression analysis to assess a moderation effect.

Unless otherwise indicated, significance level was set to α = 0.05. For explorative group comparisons and post hoc tests, *P*-values were adjusted using Benjamini–Hochberg correction and reported alongside original *P*-values.^4^ Effect sizes are interpreted according to Cohen.^12^ All analyses were performed using R.^31^ For details on the R packages used, see Supplemental Material S1 (http://links.lww.com/PR9/A273).

### 2.7. Ethics

The study, including its COVID-19 amendments, was approved by the ethics committees of Witten/Herdecke University (89/2018), the Baden-Wuerttemberg State Chamber for Medicine (B-F-2018-078), and the Faculty of Medicine at LMU Munich (18-530).

## 3. Results

### 3.1. Outcomes at LONG-TERM follow-up

At LONG-TERM, only 48% of patients reported ongoing chronic pain. The prevalence of patients with chronic pain differed between the PAC and the TAU groups (χ^2^ = 3.98; *P* = 0.046; *h* = 0.30). Although only 41% (n = 44) of the PAC group were still experiencing chronic pain, the number of patients with chronic pain in the TAU group was significantly higher (n = 57; 56%).

**Figure 2.**
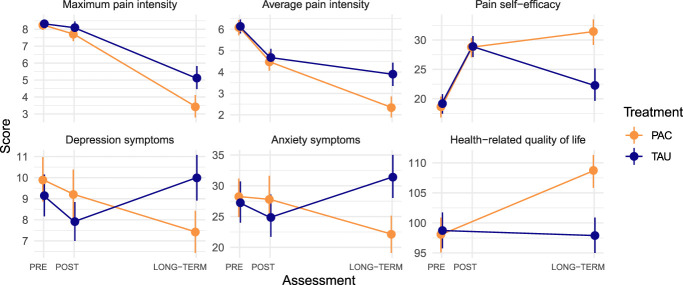
Mean trajectories of psychological parameters stratified by treatment group (psychosocial aftercare [PAC] vs treatment as usual [TAU]). Assessments took place before IIPT (PRE), at IIPT discharge (POST), and 18 to 33 months after discharge (LONG-TERM). Error bars represent 95% confidence intervals. IIPT, intensive interdisciplinary pain treatment.

Pain intensity decreased over time and differed between groups (significant MEs). A significant interaction effect showed that the reductions in average and maximum pain intensity were larger in the PAC group compared with TAU. Administering PAC led to an additional reduction of −1.62 points in maximum and −1.51 points in average pain intensity compared with TAU. For pain self-efficacy, both the MEs and interaction effects of time and treatment group were statistically significant. Implementing PAC was linked to pain self-efficacy, increasing by an additional 9.69 points compared with TAU. Concerning depression symptoms, a significant ME of time and an interaction effect emerged. Generally, there was no notable improvement in depression symptoms in the TAU group from PRE-IIPT to LONG-TERM. By contrast, PAC reduced depression symptoms by −3.63 points compared with TAU. For anxiety symptoms, only the time by treatment group interaction was significant. Compared with the TAU group, PAC corresponded to a −11.93-point reduction in anxiety symptoms. Health-related quality of life yielded significant main and interaction effects of time and treatment group. In post hoc tests, patients receiving TAU showed no significant improvement from PRE-IIPT to LONG-TERM, whereas patients with PAC improved health-related quality of life by additional 11.48 points (Fig. 2 and Table 2).

**Table 2:** Results of multilevel models including time (PRE, POST, LONG-TERM) and treatment (psychosocial aftercare and treatment as usual) for intensive interdisciplinary pain treatment outcomes.

ANOVAs	Δ*df*	Maximum pain intensity		Average pain intensity		Pain self-efficacy	
		χ^2^	*P*	χ^2^	*P*	χ^2^	*P*
Time ME	2	273.619	**<0.001**	173.886	**<0.001**	119.599	**<0.001**
Treatment ME	1	11.208	**0.001**	9.174	**0.002**	5.386	**0.020**
Interaction	2	12.937	**0.002**	16.324	**<0.001**	28.303	**<0.001**
**Post hoc tests**		**Coefficient (95% CI)**	***P***	**Coefficient (95% CI)**	***P***	**Coefficient (95% CI)**	***P***
PRE—LONG-TERM		−3.21 (−3.87 to −2.54)	**<0.001**	−2.24 (−2.82 to −1.65)	**<0.001**	3.17 (0.49 to 5.86)	**0.021**
POST—LONG-TERM		−3.00 (−3.62 to −2.39)	**<0.001**	−0.79 (−1.30 to −0.27)	**0.003**	−6.54 (−9.04 to −4.04)	**<0.001**
Treatment		−1.70 (−2.37 to −1.02)	**<0.001**	−1.57 (−2.18 to −0.95)	**<0.001**	9.15 (6.01 to 12.28)	**<0.001**
(PRE—LONG) × treatment		−1.62 (−2.54 to −0.69)	**0.001**	−1.51 (−2.33 to −0.70)	**<0.001**	9.69 (5.77 to 13.61)	**<0.001**
(POST—LONG) × treatment		−1.24 (−2.11 to −0.37)	**0.005**	−1.30 (−2.03 to −0.58)	**<0.001** *	9.23 (5.56 to 12.91)	**<0.001**

ANOVAs	Δ*df*	Depression symptoms		Anxiety symptoms		Health-related quality of life†	
		χ^2^	*P*	χ^2^	*P*	χ^2^	*P*
Time ME	2	6.008	**<0.050**	1.607	0.448	11.609	**<0.001**
Treatment ME	1	0.879	0.349	2.328	0.127	1.726	**0.001**
Interaction	2	22.002	**<0.001**	21.905	**<0.001**	16.052	**<0.001**
**Post hoc tests**		**Coefficient (95% CI)**	***P***	**Coefficient (95% CI)**	***P***	**Coefficient (95% CI)**	***P***
PRE—LONG-TERM		0.84 (−0.45 to 2.14)	0.204	4.16 (−0.07 to 8.38)	0.055	−0.83 (−4.80 to 3.13)	0.680
POST—LONG-TERM		1.96 (0.86 to 3.05)	**0.001**	6.61 (3.04 to 10.18)	**<0.001**		
Treatment		−2.57 (−4.02 to −1.12)	**0.001**	−9.27 (−14.01 to −4.53)	**<0.001**	10.84 (6.73 to 14.94)	**<0.001**
(PRE—LONG) × treatment		−3.31 (−5.12 to −1.50)	**<0.001** *	−10.27 (−16.17 to −4.37)	**0.001**	11.48 (5.94 to 17.02)	**<0.001**
(POST—LONG) × treatment		−3.63 (−5.17 to −2.08)	**<0.001**	−11.93 (−16.96 to −6.90)	**<0.001**		

Assessments took place before IIPT (PRE), at IIPT discharge (POST), and 18 to 33 months after discharge (LONG-TERM). Treatment groups were psychosocial aftercare (PAC) and treatment as usual (TAU). Reference categories were TAU for treatment; LONG-TERM was compared with the reference categories PRE-IIPT (overall treatment effect) and POST-IIPT (intervention effect). Autoregressive covariance structure is implemented for all outcomes but health-related quality of life (here, only 2 observations per patient). Number of observations per outcome (observations/patients): maximum/average pain intensity (607/209), anxiety/depression (606/209), pain self-efficacy (534/209), and health-related quality of life (418/209).

* After rounding, Benjamini–Hochberg corrected *P*-values were equivalent to unadjusted *P*-values in post hoc tests (<0.05 are set in bold), except for 2 cases indicated with an asterisk (*), where adjusted *P*-values were 0.001.

† Only 2 observations per person, thus Δdf = 1 for time ME.

Δdf, difference in degrees of freedom between compared nested models; ANOVA, analysis of variance; CI, confidence interval; IIPT, intensive interdisciplinary pain treatment; ME, main effect.

### 3.2. Impact of pandemic-related burden on treatment outcome

At LONG-TERM, 69% (n = 144) of patients reported experiencing one or more pandemic-related burdens. Only 5 patients mentioned having contracted COVID-19 themselves, 4 of which experienced at least one long-term medical consequence. Although 30% of patients had family or peers who were infected with COVID-19, there were fewer than 10 cases of hospital admissions, intensive care stays, or deaths because of COVID-19 reported. Approximately one-third of patients reported missing school because of quarantine, and one-tenth missed or rescheduled health care appointments. The incidences of financial or child care burdens in families were low overall; 22% (n = 46) of patients rated their family's financial burden as 3 or higher on an 11-point NRS, and 23% (n = 49) reported similarly for child care burdens.

Patients more frequently rated time spent with family during the pandemic positively rather than negatively. There were moderate differences between the treatment groups in terms of financial burdens during the pandemic. On average, patients who received PAC rated their family's financial burden approximately 1 point lower than patients who received TAU. Moreover, patients with PAC viewed time spent with family as significantly more relaxed than those with TAU (Table 3).

**Table 3:** Descriptive statistics of coronavirus disease 2019–related variables assessed at LONG-TERM by treatment group.

	Total sample (N = 209)	PAC (n = 107)	TAU (n = 102)	*P*	*P* (adjusted)	Cohen's (*h*/*d)*
Total pandemic-related burden*	1.33 (1.30)	1.21 (1.26)	1.46 (1.35)	0.159	0.341	−0.196
COVID-19 infection†	5 (2%)	3 (3%)	2 (2%)	1	1	0.052
COVID-19 long-term consequences†‡	4 (2%)	2 (2%)	2 (2%)	1	1	−0.007
COVID-19 family/peers	62 (30%)	34 (32%)	28 (27%)	0.594	0.990	0.110
COVID-19 hospital admission†	9 (4%)	6 (6%)	3 (3%)	0.499	0.936	0.136
COVID-19 intensive care†	5 (2%)	3 (3%)	2 (2%)	1	1	0.052
COVID-19 death†	3 (1%)	2 (2%)	1 (1%)	1	1	0.076
COVID-19 health care barriers	24 (11%)	6 (6%)	18 (18%)	0.012	0.060	−0.341
COVID-19 school absence	70 (33%)	34 (32%)	36 (35%)	0.695	1	−0.064
COVID-19 financial burden	1.64 (2.53)	1.11 (1.80)	2.20 (3.03)	0.002	**0.030**	−0.435
COVID-19 childcare difficulties	1.69 (2.64)	1.42 (2.50)	1.97 (2.77)	0.134	0.335	−0.208
Time with family						
Tense	2.01 (0.99)	1.90 (0.87)	2.14 (1.10)	0.082	0.246	−0.242
Hectic	2.07 (1.13)	2.09 (1.11)	2.05 (1.16)	0.778	1	0.039
Harmonious	2.41 (0.83)	2.54 (0.78)	2.26 (0.86)	0.015	0.056	0.339
Relaxed	2.53 (0.84)	2.69 (0.79)	2.35 (0.86)	0.004	**0.030**	0.408

Cells contain mean values (SDs) for numeric variables or absolute (relative) frequencies for dichotomous variables. χ^2^ and *t* tests were applied as appropriate (R packages *compareGroups* and *rstatix*; *P*-values withstanding Benjamini–Hochberg correction bolded). Treatment groups were psychosocial aftercare (PAC) and treatment as usual (TAU). Follow-up duration: time in days from discharge to LONG-TERM follow-up.

* Sum of COVID-19 items (range: 0–6; possible range: 0–16).

† Removed from further regression analyses because of few occurrences (n < 10).

‡ Dichotomized sum of 7 items (0 = no long-term consequences; 1 = at least one).

COVID-19, coronavirus disease 2019.

Separate LASSO regressions predicted chronic pain (lambda_1SE_ = 0.05), depression symptoms (lambda_1SE_ = 1.07), anxiety symptoms (lambda_1SE_ = 3.02), and health-related quality of life (lambda_1SE_ = 2.54) at LONG-TERM. The predictors considered were total pandemic-related burden, individual pandemic-related burdens, and ratings of time spent with family. In the model predicting chronic pain, COVID-19 infection among family members or peers (OR = 0.93) as well as pandemic-related barriers to health care access (OR = 1.60) remained as useful predictors. Specifically, patients who reported family or peers with COVID-19 were less likely to have chronic pain, whereas patients who indicated having missed or moved health care appointments because of the pandemic were more likely to have chronic pain at LONG-TERM.

By contrast, none of the pandemic-related burdens remained significant in the LASSO models predicting depression and anxiety symptoms or health-related quality of life. In these models, the ratings of time spent with family during the pandemic were predictive. Experiencing family time as tense was associated with more depression (*b* = 0.28) and anxiety symptoms (*b* = 5.03). Conversely, perceiving family time as harmonious was associated with having fewer anxiety symptoms (*b* = −1.23) and better health-related quality of life (*b* = 5.06), whereas perceiving it as relaxed was associated with less depression (*b* = −1.33) and anxiety symptoms (*b* = −2.40), as well as better health-related quality of life (*b* = 5.24). Comprehensive results of the LASSO and corresponding univariate regressions are provided in Supplemental Material S2 (http://links.lww.com/PR9/A273).

In all regression models investigating the relationship between chronic pain and psychological parameters with the total pandemic-related burden, the MEs of pandemic-related burden were not statistically significant. The moderation of burden on treatment was only significant for anxiety symptoms (interaction effect of treatment group and pandemic-related burden: *b* = 11.18, 95% CI = [1.69–20.68], *P* = 0.022; Supplemental Material S3, http://links.lww.com/PR9/A273). Although patients who received PAC had anxiety symptom scores that were, on average, 11.85 points less than those with TAU when reporting lower pandemic-related burden, they only differed by 4.56 points when patients reported higher pandemic-related burden (Supplemental Material S4, http://links.lww.com/PR9/A273).

## 4. Discussion

This study investigated whether PAC remains superior to TAU up to 2 years after PAC intervention, even amid the COVID-19 pandemic. At the LONG-TERM follow-up, which took place during the COVID-19 pandemic, the number of patients still experiencing chronic pain after IIPT was not only significantly lower for those who had received PAC compared with TAU, and they also reported lower pain intensity and higher pain-related self-efficacy. Moreover, patients receiving PAC reported higher psychological well-being overall. As hypothesized, PAC remained more effective than TAU in mitigating pain symptoms and psychological impairment—even 12 to 27 months after intervention, despite a pandemic. Psychosocial aftercare seems to help families build long-lasting resilience.

Psychosocial aftercare's advantage over TAU was smaller compared with the 12-month follow-up previously analysed.^15^ Regarding chronic pain and pain intensity, this change is likely due to the TAU group improving, thus reducing the disparity to PAC (12-month follow-up: 71% still experiencing chronic pain in TAU, 41% in PAC^15^). Although PAC patients showed significantly less psychosocial impairment than TAU patients at the 12-month follow-up, the benefit of PAC seems to have diminished during the pandemic, although it is still better compared with discharge.^15^

This study also explored the association between pandemic-related burdens and long-term treatment outcomes. This analysis was performed in 2 stages. First, differences in pandemic-related burden between PAC and TAU were explored, indicating that PAC patients reported less financial burden and perceived pandemic family time as more relaxed compared with those in the TAU group. During the 6 months of PAC, family dynamics and stressors may have been addressed, leading to higher resilience facing the pandemic. However, there were no differences between the 2 groups for other aspects of life affected by the pandemic. Second, LASSO regressions identified relevant pandemic-related predictors of treatment outcomes. Chronic pain during the pandemic correlated with a lower probability of family or peers contracting COVID-19 and more missed or rescheduled health care appointments. It could be that recovered patients lead more active social lives and thus were more likely to know people infected by COVID-19, whereas patients with persistent chronic pain might be more socially withdrawn.^19^ Furthermore, patients with chronic pain could have more health care appointments scheduled,^24^ making them more susceptible to appointment movement or cancellation during the pandemic, regardless of the intervention group.

Perceptions of family time during the pandemic predicted psychological well-being at LONG-TERM. Tense family time was associated with higher psychological impairment, whereas harmonious or relaxed family time was associated with better psychological well-being. This could be due to the reciprocal nature of the perception of social situations and one's own well-being. That is, the quality of time spent with family could influence how patients feel, and how patients feel could shape their experience of time spent with family.^18,46^ Shared variance in health-related quality of life could partly be explained by overlapping constructs, as the Kidscreen-27 contains 3 questions about the child–parent relationship.^33^

Considering all predictors simultaneously, pandemic-related burden did not predict psychological outcomes, and experiences of family time did not predict chronic pain at LONG-TERM. These findings are surprising from a biopsychosocial view of chronic pain, which posits that biological, psychological, and social factors are involved in the development and maintenance of chronic pain.^45^ The current findings, however, suggest that pandemic-related social factors might be less relevant to chronic pain recovery during the pandemic compared with other factors. One possible explanation is that experiences of time spent with family might not be directly linked to pain-related behaviours such as avoidance, neither facilitating nor preventing the use of helpful chronic pain strategies.^45^ Another explanation is that situational social factors contribute to the development of chronic pain, but are less relevant during recovery. This is supported by a longitudinal study involving schoolchildren, which found that chronic pain remission during the pandemic was not significantly predicted by pandemic-related experiences such as quality of family time, although developing chronic pain during the pandemic was significantly predicted.^32^ For former IIPT patients, changes in chronic pain induced by COVID-19 may be tied to changes in one's (often constrained) actions rather than the family environment.^28^

Finally, this study explored whether pandemic-related burden moderates PAC's treatment effect. Although pandemic-related burden alone did not predict treatment outcomes, it moderated the treatment effect on anxiety symptoms: Anxiety levels rose slightly among those who received PAC, whereas they decreased negligibly for those who received TAU. Nonetheless, those who received TAU exhibited higher anxiety than those with PAC. It can be inferred that although PAC may not fully prevent the detrimental effects of the pandemic, it does provide some alleviation. There was no difference in treatment effect based on pandemic-related burden when considering chronic pain, depression symptoms, or health-related quality of life. Similarly, another longitudinal study found that youths with chronic pain and higher personal pandemic-related burdens reported worse anxiety during the pandemic. However, they found no association with depression.^5^ The individual burden stemming from the pandemic seems to be more closely linked to anxiety symptoms rather than chronic pain, depression, or health-related quality of life. No ME of total pandemic-related burden on these outcomes was identified. Rather than being one overwhelmingly impactful factor, the COVID-19 pandemic may be part of a larger landscape of challenges affecting former pediatric IIPT patients.^27^

### 4.1. Practical implications

Psychosocial aftercare after IIPT can consolidate and potentially enhance treatment outcomes long-term, remaining effective even during adverse circumstances such as a pandemic. Although PAC cannot entirely protect families from difficult situations, it should be offered to all IIPT patients and their families to build long-lasting resilience. Psychosocial aftercare supports families long-term, regardless of their present circumstances.

### 4.2. Strengths and limitations

This study is the first to investigate the impacts of IIPT and PAC during the COVID-19 pandemic. The dropout rate in the current study is high,^43^ and factors such as experiencing high chronic pain or burden may have led to missing data, limiting generalizability of findings. However, dropout was not associated with analyzed parameters PRE-IIPT and thus is likely nonsystematic. Another limitation is that additional treatments after IIPT discharge could not be assessed objectively and were not controlled for in analyses as facilitating further treatment was part of the PAC intervention. Moreover, all pandemic-related analyses are exploratory, partly used new instruments, and revealed few significant associations, which need to be interpreted with caution.^39^ The newly created pandemic-related questions, however, cover the most relevant areas later identified by the COVID-19 Exposure Scale.^17^ LONG-TERM data are comparable across participants because they were collected shortly after the peak of the third COVID-19 wave in Germany.^8^ The large variability in LONG-TERM follow-up latency was accounted for by including follow-up duration as a control variable in all models predicting variables assessed at LONG-TERM. The current study can, therefore, be regarded as realistically capturing the experiences of young patients during the pandemic.

### 4.3. Future research

Future research could explore PAC in other settings or at earlier stages of the treatment journey. For example, at the primary care level, families could be offered support through trained social workers to navigate helpful strategies for dealing with chronic pain. Another option could involve a simpler version of PAC, such as a mobile application where families could interact with health care professionals after IIPT discharge. Furthermore, PAC could be resumed during adverse situations to further enhance and stabilize treatment success, especially regarding anxiety symptoms among both patients and parents.^5,15,22,26,38^

### 4.4. Conclusion

Life during the pandemic—marked by lockdowns, quarantines, and fluctuating school closures—was unpredictable, stressful, and frightening for children and adolescents.^1,6–8,28,34^ Even among these challenging times, PAC demonstrated its superiority over TAU up to 2 years after the intervention had ended. Psychosocial aftercare may have provided patients and their families with general resilience, although it cannot fully prevent anxiety sparked by adverse events such as a pandemic. Overall, pandemic-related experiences do not seem to have a notable impact on the treatment outcomes of former IIPT patients. Supporting families through PAC after intensive inpatient treatment should be implemented in standard health care to achieve enduring treatment effects that can withstand unforeseen adverse events. This approach can provide families with necessary support during both favourable and difficult periods.

## Disclosures

The authors have no conflicts of interest to declare.

This study was funded by the Innovation Fund of the Federal Joint Committee (grant number: 01NVF17040). The funding source had no role in the design and conduct of the study; the collection, management, analysis, and interpretation of data; the preparation, review, or approval of the manuscript; or the decision to submit the manuscript for publication. The authors report no conflicts of interest. Data and program codes are available upon request.

## Appendix A. Supplemental digital content

Supplemental digital content associated with this article can be found online at http://links.lww.com/PR9/A273.

## Supplementary Material

SUPPLEMENTARY MATERIAL
