# Supplementary material for: The effects of psychosocial aftercare following pediatric chronic pain treatment withstand the coronavirus disease 2019 pandemic: long-term outcomes of a randomized controlled trial
Source: Pain Rep. 2024 Dec 24;10(1):e1226. doi: 10.1097/PR9.0000000000001226 (PMC11671081; doi:10.1097/PR9.0000000000001226)
Supplement: SUPPLEMENTARY MATERIAL [file painreports-10-e1226-s001.pdf]

Supplemental Material S1: R Packages

- broom.mixed [2]: Benjamini-Hochberg correction
- compareGroups [11]: Group comparisons and dropout analyses.
- EMAtools [7]: Effect sizes of post-hoc tests
- finalfit [5]: Univariate regressions
- glmnet [4]: LASSO regressions
- janitor [3]: Data cleaning and descriptive statistics
- lme4 [1]: Multilevel models
- nlme [9]: Multilevel models
- psych [10]: Cronbach’s alpha
- rcompanion [8]: Nagelkerke’s pseudo R^2
- rstatix [6]: Descriptive statistics
- skimr [12]: Data examination
- tidyverse [13]: Data wrangling, analysis, and graphs

References

[1] Bates D, Mächler M, Bolker B, Walker S. Fitting Linear Mixed-Effects Models Using lme4. J Stat Soft 2015;67(1).

[2] Bolker B, Robinson D. broom.mixed: Tidying methods for mixed models, 2022.

[3] Firke S. janitor: Simple tools for examining and cleaning dirty data, 2023.

[4] Friedman J, Hastie T, Tibshirani R. Regularization paths for generalized linear models via coordinate descent. J Stat Soft 2010;33(1):1–22.

[5] Harrison E, Drake T, Ots R. finalfit: Quickly create elegant regression results tables and plots when modelling, 2023.

[6] Kassambara A. rstatix: Pipe-friendly framework for basic statistical tests, 2020.

[7] Kleiman E. EMAtools: Data management tools for real-time monitoring/ecological momentary assessment data, 2021.

[8] Mangiafico S. rcompanion: Functions to support extension education program evaluation, 2020.

[9] Pinheiro J, Bates D, DebRoy S, Sarkar D, R Core Team. nlme: Linear and nonlinear mixed effects models, 2020.

[10] Revelle W. psych: Procedures for psychological, psychometric, and personality research. Evanston, Illinois, 2023.

[11] Subirana I, Sanz H, Vila J. Building bivariate tables: The compareGroups package for R. J Stat Soft 2014;57(12):1–16.

[12] Waring E, Quinn M, McNamara A, Eduardo Arino de la Rubia, Zhu H, Ellis S. skimr: Compact and flexible summaries of data, 2022.

[13] Wickham H, Averick M, Bryan J, Chang W, McGowan LD, François R, Grolemund G, Hayes A, Henry L, Hester J, Kuhn M, Pedersen TL, Miller E, Bache SM, Müller K, Ooms J, Robinson D, Seidel DP, Spinu V, Takahashi K, Vaughan D, Wilke C, Woo K, Yutani H. Welcome to the tidyverse. J Open Source Soft 2019;4(43):1686.

Table S2

Univariate regression and LASSO regression results for chronic pain and psychological outcomes at LONG-TERM

**Chronic pain**	OR	95% CI [L; U]		*p*	R^2	LASSO
*Gender (girl) **	1.43	[0.77;	2.68]	.262	.008	1.41
Age	1.25	[0.73;	2.17]	.423	.004	1.22
*Treatment (PAC) **	0.55	[0.32;	0.95]	**.033**	.029	0.58
Follow-up duration	1.42	[0.82;	2.47]	.210	.010	1.50
Total pandemic-related burden	0.91	[0.52;	1.56]	.722	.001	–
*COVID-19 family/peers **	0.47	[0.25;	0.87]	**.017**	.037	0.93
*COVID-19 school absence **	0.66	[0.37;	1.17]	.158	.013	–
*COVID-19 healthcare barriers **	6.42	[2.32;	22.73]	**.001**	.088	1.60
COVID-19 financial burden	2.19	[1.24;	4.06]	**.009**	.047	–
COVID-19 child care difficulties	0.94	[0.54;	1.61]	.810	<.001	–
Time with family						
tense	1.57	[0.91;	2.76]	.108	.017	–
hectic	1.35	[0.78;	2.34]	.286	.007	–
harmonious	0.54	[0.30;	0.93]	**.030**	.031	–
relaxed	0.47	[0.26;	0.82]	**.009**	.045	–

**Depressive symptoms**	*b*	95% CI [L; U]		*p*	R^2	LASSO
*Gender (girl) **	2.27	[0.57;	3.98]	.009	.032	2.17
Age	0.51	[-1.02;	2.03]	.515	.002	0.21
*Treatment (PAC) **	-2.57	[-4.05;	-1.08]	**.001**	.053	-2.21
Follow-up duration	1.21	[-0.31;	2.73]	.119	.012	1.20
Total pandemic-related burden	1.06	[-0.46;	2.59]	.170	.009	–
*COVID-19 family/peers **	-0.57	[-2.24;	1.10]	.502	.002	–
*COVID-19 school absence **	0.60	[-1.02;	2.21]	.467	.003	–
*COVID-19 healthcare barriers **	2.06	[-0.31;	4.44]	.089	.014	–
COVID-19 financial burden	2.37	[0.87;	3.86]	**.002**	.045	–
COVID-19 child care difficulties	0.71	[-0.82;	2.24]	.361	.004	–
Time with family						
tense	4.23	[2.82;	5.65]	**<.001**	.144	0.28
hectic	2.21	[0.71;	3.71]	**.004**	.039	–
harmonious	-4.27	[-5.68;	-2.86]	**<.001**	.146	–
relaxed	-5.18	[-6.53;	-3.82]	**<.001**	.215	-1.33

**Anxiety symptoms**	*b*	95% CI [L; U]		*p*	R^2	LASSO
*Gender (girl) **	4.54	[-1.04;	10.12]	.110	.012	4.55
Age	-0.17	[-5.13;	4.79]	.947	<.001	-1.25
*Treatment (PAC) **	-9.27	[-14.05;	-4.49]	**<.001**	.066	-7.86
Follow-up duration	4.09	[-0.84;	9.01]	.103	.013	3.85
Total pandemic-related burden	2.72	[-2.23;	7.66]	.280	.006	–
*COVID-19 family/peers **	-2.95	[-8.35;	2.45]	.283	.006	–
*COVID-19 school absence **	-1.16	[-6.39;	4.08]	.663	.001	–
*COVID-19 healthcare barriers **	5.05	[-2.67;	12.77]	.199	.008	–
COVID-19 financial burden	10.35	[5.61;	15.10]	**<.001**	.082	–
COVID-19 child care difficulties	4.47	[-0.44;	9.39]	.074	.015	–
Time with family						
tense	16.17	[11.73;	20.60]	**<.001**	.200	5.03
hectic	9.90	[5.13;	14.66]	**<.001**	.075	–
harmonious	-14.68	[-19.21;	-10.15]	**<.001**	.165	-1.23
relaxed	-16.10	[-20.54;	-11.66]	**<.001**	.198	-2.40

Health-related quality of life	*b*	95% CI [L; U]		*p*	R^2	LASSO
*Gender (girl) **	-6.99	[-11.94;	-2.04]	**.006**	.036	-7.02
Age	-0.94	[-5.39;	3.51]	.678	.001	0.27
*Treatment (PAC) **	10.84	[6.65;	15.02]	**<.001**	.112	8.76
Follow-up duration	-5.05	[-9.45;	-0.65]	**.025**	.024	-4.63
Total pandemic-related burden	-1.89	[-6.33;	2.56]	.404	.003	–
*COVID-19 family/peers **	3.12	[-1.72;	7.97]	.205	.008	–
*COVID-19 school absence **	-0.14	[-4.85;	4.57]	.954	<.001	–
*COVID-19 healthcare barriers **	-6.96	[-13.86;	-0.06]	**.048**	.019	–
COVID-19 financial burden	-6.76	[-11.11;	-2.40]	**.003**	.002	–
COVID-19 child care difficulties	-1.20	[-5.65;	3.25]	.594	.001	–
Time with family						
tense	-7.38	[-11.72;	-3.05]	**.001**	.052	–
hectic	-1.86	[-6.31;	2.58]	.410	.003	–
harmonious	16.92	[13.11;	20.72]	**<.001**	.271	5.06
relaxed	18.26	[14.58;	21.94]	**<.001**	.316	5.24

Notes. Displayed are coefficients (Odds Ratio = OR; unstandardized coefficient = *b*), 95% confidence intervals (CI), *p*-values, and Nagelkerke's pseudo-R^2. Logistic regressions were used for chronic pain (0 = no chronic pain; 1 = chronic pain). For all other outcomes, linear regressions were used. LASSO = least absolute shrinkage and selection operator; displayed for LASSO regressions are the unstandardized coefficients of predictors that remained in the model. *p*-values < .05 are bolded. L = lower boundary; U = upper boundary. Nagelkerke's pseudo-R^2 was calculated using the R package *rcompanion*.

* For dichotomous predictors, “no” was reference category unless otherwise indicated.

Table S3

Multivariate regressions investigating association of total pandemic-related burden with chronic pain and psychological outcomes at LONG-TERM

**Chronic pain**	OR	95% CI [L; U]		*p*
*Gender (girl) **	1.41	[0.74;	2.70]	.295
Age	1.25	[0.71;	2.22]	.435
*Treatment (PAC) **	0.53	[0.30;	0.93]	**.028**
Follow-up duration	1.53	[0.87;	2.71]	.142
Total pandemic-related burden	0.83	[0.38;	1.82]	.633
Burden x Treatment Interaction	0.94	[0.30;	2.90]	.910

**Depression symptoms**	*b*	95% CI [L; U]		*p*
*Gender (girl) **	2.36	[0.69;	4.02]	**.006**
Age	0.30	[-1.16;	1.77]	.685
*Treatment (PAC) **	-2.44	[-3.90;	-0.99]	**.001**
Follow-up duration	1.46	[-0.01;	2.93]	.052
Total pandemic-related burden	-0.48	[-2.51;	1.56]	.648
Burden x Treatment Interaction	2.44	[-0.49;	5.38]	.104

**Anxiety symptoms**	*b*	95% CI [L; U]		*p*
*Gender (girl) **	5.08	[-0.31;	10.47]	.066
Age	-0.38	[-5.13;	4.36]	.874
*Treatment (PAC) **	-9.01	[-13.72;	-4.31]	**< .001**
Follow-up duration	4.91	[0.16;	9.66]	**.044**
Total pandemic-related burden	-3.90	[-10.50;	2.70]	.248
Burden x Treatment Interaction	11.18	[1.69;	20.68]	**.022**

**Health-related quality of life**	*b*	95% CI [L; U]		*p*
*Gender (girl) **	-7.20	[-11.87;	-2.54]	**.003**
Age	-0.47	[-4.58;	3.64]	.823
*Treatment (PAC) **	10.64	[6.57;	14.71]	**< .001**
Follow-up duration	-5.95	[-10.06;	-1.84]	**.005**
Total pandemic-related burden	2.23	[-3.48;	7.94]	.444
Burden x Treatment Interaction	-5.34	[-13.56;	2.87]	.204

Notes. Displayed are coefficients (Odds Ratio = OR; unstandardized coefficients = *b*), 95% confidence intervals (CI), and *p*-values. Logistic regressions were used for chronic pain (0 = no chronic pain; 1 = chronic pain). For all other outcomes, linear regressions were used. *p*-values < .05 are set in bold. L = lower boundary; U = upper boundary.

* Reference categories for dichotomous predictors were TAU for treatment and boy for gender.

Table S4

Means (standard deviations) of chronic pain and psychological outcomes at LONG-TERM by total pandemic-related burden (low vs. high) and treatment group (PAC vs. TAU)

	**Low total pandemic-related burden**		**High total pandemic-related burden**	
	PAC<br>*n* = 72	TAU<br>*n* = 60	PAC<br>*n* = 35	TAU<br>*n* = 42
Chronic pain *	0.44 (0.50)	0.57 (0.50)	0.34 (0.48)	0.55 (0.50)
Depression symptoms	7.18 (4.61)	10.12 (5.43)	7.91 (6.54)	9.81 (5.88)
Anxiety symptoms	20.50 (14.30)	32.35 (18.75)	25.49 (18.91)	30.05 (19.39)
Health-related quality of life	109.03 (15.49)	97.82 (15.86)	108.14 (15.08)	98.02 (14.94)

Notes. PAC = Psychosocial aftercare; TAU = treatment as usual; LONG-TERM = follow-up assessment 18 to 33 months after discharge. High total pandemic-related burden = score larger than mean; low total pandemic-related burden = score smaller than or equal to mean.

* 0 = no chronic pain; 1 = chronic pain.
